# The HIV infection and telephone counseling, the experience of Italian National Institute of Health

**DOI:** 10.1186/1742-4690-9-S1-P124

**Published:** 2012-05-25

**Authors:** Anna Maria Luzi, Anna Colucci, Filippo Maria Taglieri, Pietro Gallo, Rudi Valli, Francesca Botta, Eleonora Lichtner

**Affiliations:** 1 I.S.S., Rome, Italy

## Introduction

In the context of HIV prevention interventions, counseling has proved a valuable operational tool. This method is characterized by the application of knowledge, personal qualities, skills (active listening, self-awareness, empathy) and communication techniques (reformulation, clarification, investigation skills). The interview give the person counseling-user to make choices and changes in situations perceived as difficult to deal with his problems in an active way and its difficulties. The above constitutes the methodological basis of HIV / AIDS Helpline telephone counseling done by the National Institute of Health, established in 1987.

## Materials and methods

The intervention of telephone counseling provided by AIDS HELP LINE Italian is structured in three phases: initial, intermediate and final. The actions that characterize the three phases of the telephone counseling are as follows:

- Accommodating the person / service user and present the Service

- To focus the problem and find a shared goal

- Provide information scientifically accurate, current and customized

- Propose and agree on possible solutions

- Summarise and check what has emerged and what has been agreed

- Saying goodbye properly, make themselves available for further contacts and enter into the relationship

The Italian AIDS Helpline uses a data-entry software that allows you to collect data on calls received. The data are handled anonymously and analyzed in aggregate form.

## Results

People - users in over 24 years, have turned to the AIDS Helpline Italian ISS are 671,823, of these 73.9% were males aged between 20-39 years (78.0%), reside in Central Italy, declare themselves heterosexual (55.1%) and raise questions regarding information on the procedures for transmitting HIV (27.2%) and ITER diagnostics (25.3%).

## Conclusions

The intervention of telephone counseling conducted by the AIDS Helpline Italian while placing no longer in a state of emergency and as a social alarm in the '80s, however, the data show that a significant number of people are taking advantage of telephone counseling intervention to express their information needs, clarify doubts and get information about the psycho-social-health services on national territory involved in the prevention, diagnosis and treatment of AIDS.

